# Identification of multiple novel susceptibility genes associated with autoimmune thyroid disease

**DOI:** 10.3389/fimmu.2023.1161311

**Published:** 2023-05-01

**Authors:** Xueying Liu, Yahu Miao, Chao Liu, Wan Lu, Qing Feng, Qiu Zhang

**Affiliations:** Department of Endocrinology, First Affiliated Hospital of Anhui Medical University, Hefei, China

**Keywords:** autoimmune thyroid disease, GWAS, TWAS, FUMA, SMR

## Abstract

**Background:**

Autoimmune thyroid disease (AITD) is induced by various factors, including inheritability, which regulates gene expression. Multiple loci correlated with AITD have been discovered utilizing genome-wide association studies (GWASs). Nevertheless, demonstrating the biological relevance and function of these genetic loci is difficult.

**Methods:**

The FUSION software was utilized to define genes that were expressed differentially in AITD using a transcriptome-wide association study (TWAS) method in accordance with GWAS summary statistics from the largest genome-wide association study of 755,406 AITD individuals (30,234 cases and 725,172 controls) and levels of gene expression from two tissue datasets (blood and thyroid). Further analyses were performed such as colocalization, conditional, and fine-mapping analyses to extensively characterize the identified associations, using functional mapping and annotation (FUMA) to conduct functional annotation of the summary statistics of 23329 significant risk SNPs (*P* < 5 × 10^−8^) recognized by GWAS, together with summary-data-based mendelian randomization (SMR) for identifying functionally related genes at the loci in GWAS.

**Results:**

There were 330 genes with transcriptome-wide significant differences between cases and controls, and the majority of these genes were new. 9 of the 94 unique significant genes had strong, colocalized, and potentially causal correlations with AITD. Such strong associations included *CD247*, *TPO*, *KIAA1524*, *PDE8B*, *BACH2*, *FYN*, *FOXK1*, *NKX2-3*, and *SPATA13*. Subsequently, applying the FUMA approach, novel putative AITD susceptibility genes and involved gene sets were detected. Furthermore, we detected 95 probes that showed strong pleiotropic association with AITD through SMR analysis, such as *CYP21A2*, *TPO*, *BRD7*, and *FCRL3*. Lastly, we selected 26 genes by integrating the result of TWAS, FUMA, and SMR analysis. A phenome-wide association study (pheWAS) was then carried out to determine the risk of other related or co-morbid phenotypes for AITD-related genes.

**Conclusions:**

The current work provides further insight into widespread changes in AITD at the transcriptomic level, as well as characterized the genetic component of gene expression in AITD by validating identified genes, establishing new correlations, and uncovering novel susceptibility genes. Our findings indicate that the genetic component of gene expression plays a significant part in AITD.

## 1 Introduction

Autoimmune thyroid disease (AITD), which comprises Graves’ disease (GD) and Hashimoto’s thyroiditis (HT), is induced by an immune system dysfunction that results in an immune response on the thyroid. It is one of the most typical autoimmune conditions, which is genetically heterogeneous (1). According to epidemiological data regarding genetic susceptibility to AITD, AITD concordance is greater than 50% in monozygotic twins (2). The vast majority of AITD cases go undetected until the immunological response damages the thyroid gland. This is in complete contradiction to other prevalent autoimmune conditions that frequently co-occur with AITD (3), such as rheumatoid arthritis (RA), where several risk loci have been found, multiple effective treatments are obtainable, and an effort is being made on early detection and even disease prevention (4, 5). With advances in comprehending the immunological and molecular basis of autoimmune conditions, gene therapy has emerged as a potential treatment option for patients who suffer from the disease (6). Therefore, the demand for prevention and early treatment of AITD is necessary, as well as a thorough knowledge of disease pathogenesis.

Genome-wide association studies (GWASs) have been frequently used, and they constitute an important step in identifying the particular genes that underlie AITD genetic risk. Six loci with evidence of AITD linkage were revealed in a genome-wide study of 56 multigenerational AITD families (354 people) utilizing 387 microsatellite markers (7). In addition, Chu et al. (8) performed a GWAS in 1,536 patients with GD (cases) and 1,516 controls and then examined a group of associated single nucleotide polymorphisms (SNPs) in a second sample of 3,994 cases and 3,510 controls. They identified two novel susceptibility loci and corroborated four previously described loci (in the major histocompatibility complex, *TSHR*, *CTLA4*, and *FCRL3*) (the *RNASET2-FGFR1OP-CCR6* region at 6q27 and an intergenic region at 4p14). Oryoj et al. (9) discovered a new SNP at the *VAV3* locus associated with HT using a sample of 181 cases and 1363 healthy controls. Even though GWAS has allowed the discovery of SNPs that confer susceptibility to AITD, the functional significance of these genetic variants is unknown. Examining intermediary processes between the genome and the phenotype, like gene expression, may assist us in comprehending the molecular mechanisms behind AITD.

TWAS, one method for prioritizing causative genes at GWAS loci, could use expression reference panels (eQTL cohorts with expression and genotype data) to identify gene-trait associations from GWAS datasets (10–12). Furthermore, Zhu et al. (11) introduced Summary-data-based Mendelian Randomization (SMR), which may incorporate summary-level data from GWAS with eQTL data to find genes related to a complex trait due to pleiotropy at the loci found in GWAS. Using this method, previous research has recently discovered novel trait-associated genes for 33 human complex traits (11, 13). To date, these methods applied to AITD have not been well documented.

Herein, we carried out a TWAS to find genetically regulated genes correlated with AITD. Using conditional analysis, we revealed that genetically regulated expression generates several genome-wide significant signals from the AITD GWAS. Then we investigate how these genes induce dysregulation of neighboring co-expressed genes. Furthermore, Functional Mapping and Annotation (FUMA) was performed to make a functional annotation for significant risk SNPs identified by GWAS. SMR analysis was then employed to identify significant genes that were pleiotropically related to AITD. Finally, we performed a PheWAS and identified the risk of other associated or co-morbid phenotypes for AITD-related genes. This present research will advance the field by elucidating the molecular mechanisms of AITD, including several disease-relevant tissues, and identifying critical genes for the disease that might be future prospects for investigation on its therapy and etiology.

## 2 Materials and methods

### 2.1 Datasets

The analysis used 1) genome-wide summary statistics from the GWAS of AITD by Saevarsdottir et al. (14), 2) 4 SNP weight sets from 3 separate transcriptomic reference samples (GTEx v8, NTR (Netherlands Twin Register) and YFS (Young Finns Study), and 3) the 1000 Genomes Project linkage disequilibrium (LD) reference.

To begin, we obtained the GWAS data from 755,406 European participants, including 30,234 cases and 725,172 controls, and the AITD summary statistic from the deCODE Genetics SUMMARY DATA (14), containing genetic susceptibility information to AITD for 36,004,550 HapMap3 SNPs from two datasets, Iceland and the UK Biobank.

In addition, SNP weights from three tissues were utilized: peripheral blood, whole blood, and thyroid. SNP weights indicate the associations between SNPs and the expression of their associated gene. Four SNP weight sets were obtained from the TWAS FUSION official website (http://gusevlab.org/projects/fusion/#reference-functional-data). The weights were assigned to four RNA reference samples as follows: The Netherlands Twin Register (NTR) and the Young Finns Study (YFS) both yield gene expression data on blood tissue, and the GTEx Consortium, a cutting-edge study that examines expression in whole blood and thyroid separately. Finally, the FUSION website was used to obtain the 1000 Genomes Phase 3 European LD reference, which included 489 individuals (http://gusevlab.org/projects/fusion/).

### 2.2 Statistical analysis

Ubuntu 22/Bash (GNU Project Bourne Again Shell) and R version 4.2.1 (The R Project for Statistical Computing, Vienna, Austria Anhui, China) were utilized to conduct all statistical analyses.

### 2.3 Transcriptome-wide significance threshold

To calculate the transcriptome-wide significance threshold for this investigation, we employed a permutation approach from a previous TWAS (15). This method calculates a significant threshold that is modified for the number of evaluated features and takes feature correlation within and across SNP weight sets into account. A strict Bonferroni-corrected study-wise threshold was set: *P* = 2.38 × 10^-6^ (0.05/21,053) (total quantity of genes across panels).

### 2.4 TWAS FUSION and colocalization

FUSION is a series of programs for carrying out transcriptome-wide and regulome-wide association studies (TWAS and RWAS). FUSION develops predictive models of the genetic component of a functional/molecular phenotype and analyzes disease-associated components using GWAS summary statistics (16). TWAS analysis was carried out in this study utilizing software that followed the TWAS FUSION protocol with default settings (16). In addition, the colocalization test, which gives data of a shared causal variant between the predicted functional feature and the trait, would be an alternative to the TWAS test (which looks for a substantial correlation between the predicted functional feature and the trait). Colocalization was conducted using the coloc R package with a 0.5Mb window for all genes meeting transcriptome-wide significance (*P* < 0.05), and FUSION was used to evaluate the posterior probability (PP) that GWAS and TWAS associations share a causal SNP. The coloc statistic for each of the coloc hypotheses is represented by the PP0 (no association), PP1 (functional association only), PP2 (GWAS association only), PP3 (independent functional/GWAS associations), and PP4 (colocalized functional/GWAS associations) columns.

### 2.5 Conditional analysis

Using the FUSION software, conditional and joint analyses were conducted for transcriptome-wide significant (Bonferroni-corrected criteria) signals to confirm whether these significant signals were attributable to multiple-associated features or conditionally independent (16). The software assesses the influence of all important features within each locus by examining residual SNP correlations with AITD after accounting for the predicted expression of other features (16). When other features in the region’s predicted expression are taken into consideration, the approach may identify which features indicate independent associations, referred to as jointly significant, and which do not, referred to as marginally significant (16). For post-processing and creating numerous conditional output plots as well as summary statistics, the “FUSION.post process.R” script was utilized.

### 2.6 Statistical fine mapping

Mancuso N et al. (17) introduced FOCUS, a fine-mapping method that may estimate credible sets of causal genes from TWAS utilizing prediction eQTL weights, LD, and GWAS summary statistics. FOCUS assesses the posterior inclusion probability (PIP) of each feature being causal in a region of association, similar to statistical fine mapping of GWAS results, and uses the sum of PIP to construct the default 90% credible set, a set of features expected to contain the causal features. The PIP of individual features with PIP > 0.5 indicates that a feature has a higher probability of being causal than any other feature in the region. Without using the tissue prioritization option, the FOCUS fine mapping function was performed on all SNP weight panels at the same time.

### 2.7 Functional mapping and annotation of GWAS

#### 2.7.1 Definition of genomic risk loci based on GWAS

The AITD meta-analysis, which included 30,234 cases and 725,172 controls, identified 23,329 SNPs with *P* < 5.0 × 10^-8^ (14). For identifying genomic risk loci for AITD susceptibility according to previous GWAS summary statistics (14), LD structure from the European ancestry of the 1000 Genomes Reference panel was computed and utilized in FUMA (18). Based on previous literature, genomic risk loci, independent significant SNPs, lead SNPs, and candidate SNPs were defined (18).

#### 2.7.2 Annotation of candidate SNPs in genomic risk loci

FUMA is a post-GWAS annotation program that uses candidate SNPs to identify possible causal variants for AITD. The FUMA platform was used to develop a number of SNP functional annotation approaches (18). ANNOVAR (19) (‘gene-based annotation’) is used to examine the functional consequences of all SNPs on genes utilizing Ensembl genes (build 85). Note that candidate SNPs were annotated for functional consequences (3′ untranslated region, 5′ untranslated region, downstream, exonic, intergenic, intronic, ncRNA_exonic, ncRNA_intronic, ncRNA_splicing, splicing, upstream across genes) on gene functions.

#### 2.7.3 MAGMA for gene analysis and gene-set analysis

FUMA utilizes the MAGMA tool to generate gene-based *P*-values (gene analysis) and gene set *P*-values (gene set analysis) from input AITD GWAS summary statistics (20). The major histocompatibility complex region and indels were eliminated from the analysis. For gene analysis, all SNPs within genes were mapped to 19,987 protein-coding genes. The default significant genes threshold is defined as *P* = 2.50 × 10^−6^ (the Bonferroni correction, 0.05/19,987). Individual genes that share particular biological activities were pooled and studied for correlation with disease or trait in gene set analysis. The gene set *P*-value is calculated utilizing the same threshold criteria (*P* = 0.05/15,488).

### 2.8 SMR analysis

SMR analyses were carried out to observe genes that were causally associated with AITD, allowing us to prioritize functionally relevant genes in the GWAS loci (11). SMR incorporates GWAS and eQTL summary statistics to assess for a pleiotropic correlation between gene expression and a trait owing to a shared variant at a locus using Mendelian Randomization (MR) principles.

The cis-eQTL genetic variants were utilized as the instrumental variables (IVs) for gene expression in the SMR analysis. For gene expression in blood and thyroid, SMR analysis was conducted separately. Meanwhile, for blood, we employed the Westra, CAGE and GTEx v8 eQTL summarized data (21–23), which comprised 3,511, 2,765, and 670 participants respectively. In addition, we utilized the version 8 release of the GTEx eQTL summarized data (23), which was for thyroid tissue with 574 participants. The blood and thyroid eQTL data may be downloaded online (https://yanglab.westlake.edu.cn/software/smr/#DataResource).

To assess the presence of LD in the identified association, the heterogeneity in dependent instruments (HEIDI) test was conducted. The genome-wide significance level for the SMR test is defined as 0.05/the number of probes. The significance values of these probes are *P* < 5.89 × 10^-6^ (peripheral blood from CAGE.sparse), *P* < 8.44 × 10^-6^ (peripheral blood from westra_eqtl_hg19), *P* < 7.65 × 10^-6^ (whole blood from GETx), and *P* < 5.57 × 10^-6^ (thyroid from GETx) respectively and those probes with little evidence of heterogeneity (*P*_HEIDI_ ≥ 0.05) were retained.

### 2.9 Phenome-wide association studies

A phenome-wide association study (PheWAS) was undertaken utilizing publicly accessible data from the GWAS Atlas (https://atlas.ctglab.nl) to identify phenotypes associated with the AITD genes *via* TWAS. The top ten phenotypes were reported.

## 3 Result

### 3.1 Transcriptome-wide significant hits

**Figure 1:**
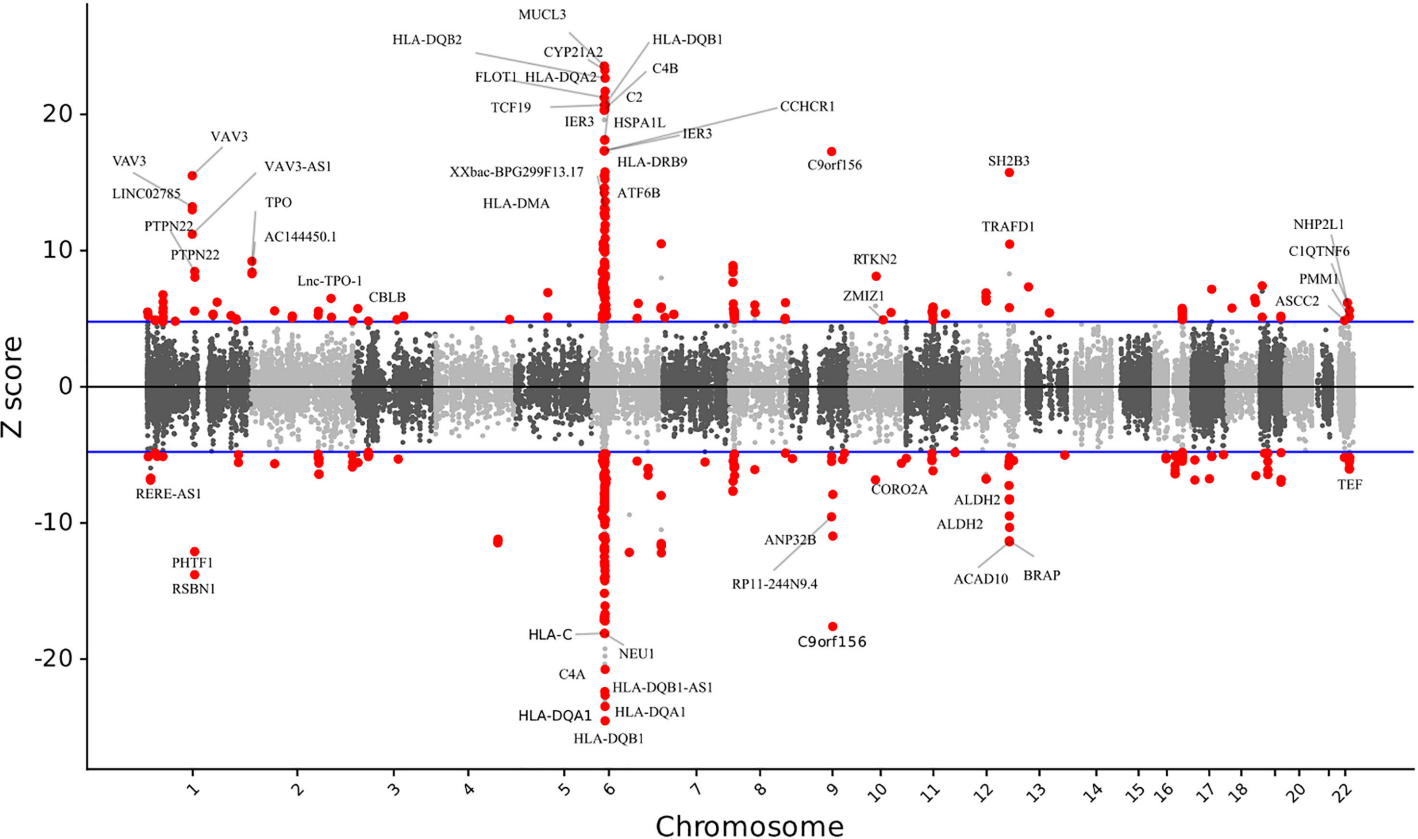
The relationship between gene expression and autoimmune thyroid disease. Manhattan-style plot of z scores for each of the tested genes, across all autosomes and tested single nucleotide polymorphism weight sets. Blue lines indicate the transcriptome-wide significance threshold. All the statistically significant genes are shown.

We discovered 499 significant features from 330 unique genes that were differentially expressed (*P* < 2.38 × 10^-6^) across several SNP weight sets in AITD (**Figure 1** **;** **Supplementary Table S1**). Among the 499 significant features, 237 were upregulated, while 262 were downregulated. In comparison to the previous AITD GWAS by Saevarsdottir S et al, Chu et al, and Oryoji et al. (8, 9, 14), 296 unique genes were novel and 34 were previously implicated. The largest number of associations were from the GTEx thyroid set (209 associated features).

### 3.2 Colocalization analysis

We calculated the PP that the genetic and functional correlations generated by various causal SNPs (PP3) and a shared causal SNP (PP4) to evaluate the colocalization status of the gene. 159 (118 unique genes) of the 499 significant features were regarded as colocalized based on their high PP4 content (PP4 > 0.8). It was observed that 12 significant features (9 unique genes) explain all of the signals at their loci (PP4 = 1) **(** **Supplementary Table S2** **)**.

### 3.3 Conditional analysis

We discovered that numerous significant features were located inside the same locus (defined as a 0.5Mb window). Conditional analysis of the 499 significant features revealed 135 jointly (129 unique genes) and 364 marginally significant features (**Supplementary Table S3**), indicating that many identified genes were linked to AITD owing to their co-expression with the 135 independent features.

We further examined how adjusting for gene expression in features impacted the correlations between SNPs and traits. The percentage of variance in GWAS correlations accounted for gene expression correlations at a specific location ranges from 4.94% to 100%. Genome-wide association signals on Chromosomes 1, 2, 4, 8, 11, 12, 13, 16, 17, 18 and 22 fully explained relative genetic variations (**Supplementary Table S4**). This might imply that genetic risk for AITD is mediated by functional feature alterations at these loci.

### 3.4 TWAS fine mapping

FOCUS was conducted to compute PIP for each feature (17). We found 16 significant features (16 unique genes) with PIP > 0.5 in three tissues, suggesting that these are likely causal in their correlations with AITD. Of these, 9 genes were supported by the colocalization analysis (**Table 1**). The genes with the highest possibility of causality were *SH2B3* (YFS blood), *SPATA13* (GTEx thyroid), *TPO* (GTEx thyroid), *IRF4* (GTEx thyroid), and *BACH2* (NTR blood) (PIP = 1.00) (**Table 1**). Furthermore, we identified multiple significant features from other tissues, such as adipose, artery, brain, liver, and so forth. The detailed information is presented in **Supplementary Table S5**.

**Table 1:** Causal posterior probabilities for genes in 90%-credible sets for AITD TWAS signals.

Gene ID	Gene set	twas_z	PIP	Region	Colocalized
*CAPZB*	GTEx Thyroid	5.03	0.718	1:18663253-20469098	No
*CD247*	YFS Blood	5.91	0.973	1:166460527-169086221	Yes
*TPO*	GTEx Thyroid	9.25	1.000	2:10188-1781022	Yes
*KIAA1524*	GTEx Whole_blood	-5.05	0.638	3:106983112-109522395	Yes
*PDE8B*	GTEx Thyroid	6.72	0.998	5:75798866-77622868	Yes
*IRF4*	GTEx Thyroid	6.78	1.000	6:100116-1452209	No
*BACH2*	NTR Blood	-11.50	1.000	6:89973052-91842836	Yes
*FYN*	YFS Blood	5.79	0.999	6:110304247-112344737	Yes
*FOXK1*	GTEx Whole_blood	5.31	0.559	7:4573890-5416232	Yes
*SGK223*	GTEx Thyroid	8.50	0.851	8:7153384-9154694	No
*RTKN2*	NTR Blood	8.05	0.986	10:63341695-65793994	No
*NKX2-3*	GTEx Thyroid	5.81	0.919	10:100668400-102948934	Yes
*CD44*	NTR Blood	-5.00	0.960	11:33958739-35502340	No
*SH2B3*	YFS Blood	15.50	1.000	12:110336719-113263518	No
*SPATA13*	GTEx Thyroid	7.97	1.000	13:24531937-25784362	Yes
*CTB-41I6.2*	GTEx Whole_blood	5.10	0.790	17:8307571-9965921	No

GTEx, Genotype-Tissue expression; NTR, Netherlands Twin Register; YFS, Young Finns Study; PIP, the posterior inclusion probability. Highlighted genes are PIP=1.

### 3.5 FUMA of GWAS

#### 3.5.1 Functional annotation analysis by FUMA

**Figure 2:**
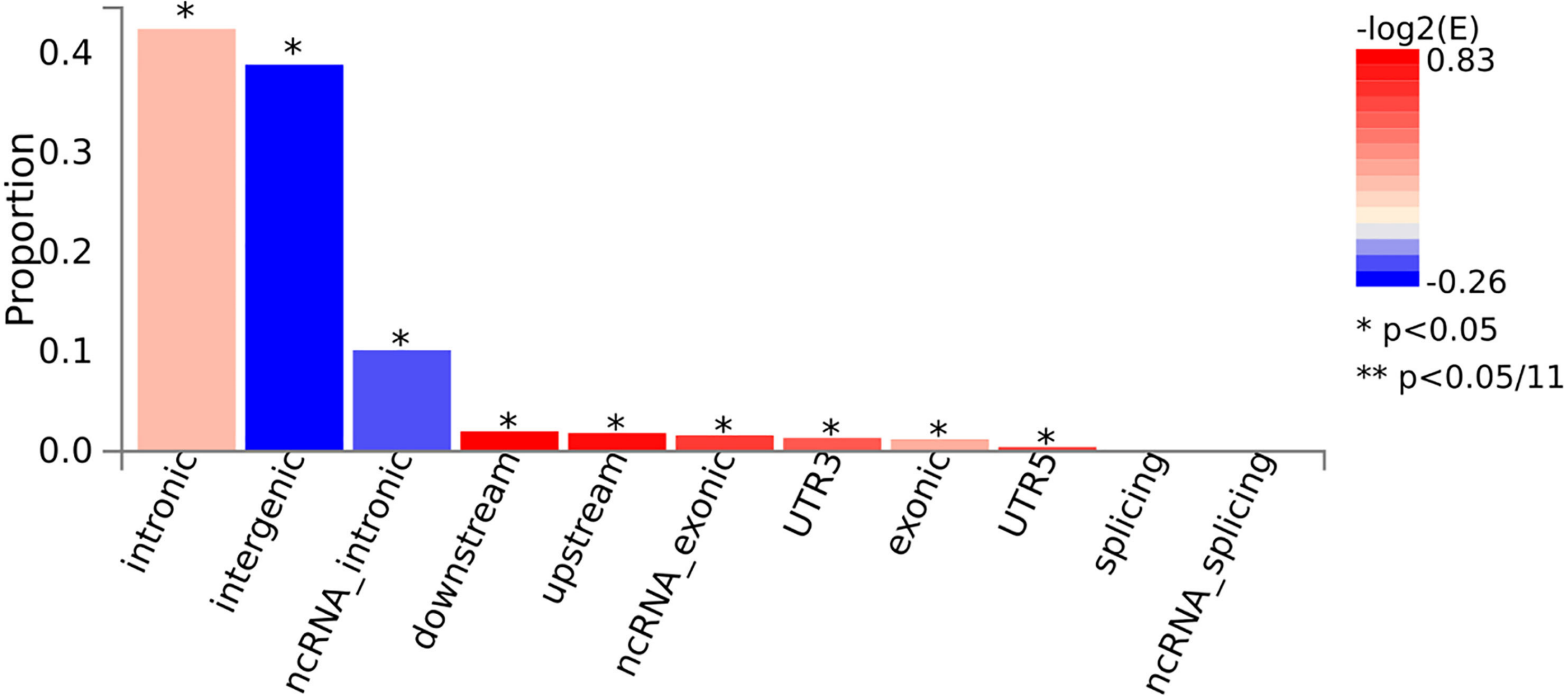
The consequences of candidate SNPs functionally annotated. *P < 0.05.

The FUMA approach was utilized to conduct functional annotation of AITD GWAS summary statistics, which included 30,234 cases and 725,172 controls. Starting with the AITD GWAS summary statistics, 259 lead SNPs and 23,329 candidate SNPs with LD with the lead SNPs were discovered among 955 independent significant SNPs across 128 genomic loci (**Figure 2** **;** **Supplementary Figure 1**). We totally prioritized 583 unique genes from 128 loci through FUMA (**Supplementary Table S6**, **S7**).

#### 3.5.2 Gene analysis, gene-set analysis

There were 424 significant genes found (**Supplementary Table S8**), and 15,488 gene sets were identified. 142 of these gene sets were found to be significant (*P* < 3.23 ×10^-6^). The most significant gene set was positive regulation of the immune system process (*P* = 7.18×10^-22^), followed by regulation of the immune system process (*P* = 1.19 × 10^-22^). Of these pathways, 50 gene sets associated with AITD were tested, and 159 were associated with other autoimmune conditions such as systemic lupus erythematosus (SLE) and type 1 diabetes mellitus (T1DM) (**Supplementary Table S9**).

### 3.6 SMR analysis

#### 3.6.1 Basic information of the summarized data

According to the SMR analysis, The number of probes was 20,943 (8485 from CAGE.sparse, 5923 from westra_eqtl_hg19, and 6535 from GETx whole blood) and 8,972 in the analysis for blood and thyroid, respectively.

#### 3.6.2 SMR analysis in blood

**Figure 3:**
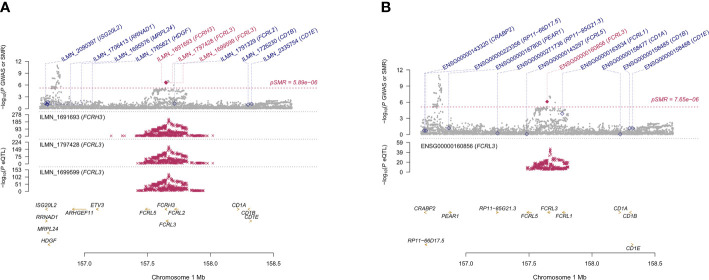
Prioritizing genes around *FCRL3/FCRH3* in association with AITD in blood. Top plot, gray dots represent the -log 10 (*P* values) for SNPs from the GWAS of AITD, and rhombuses represent the -log 10 (*P* values) for probes from the SMR test with solid rhombuses indicating that the probes pass HEIDI test and hollow rhombuses indicating that the probes do not pass the HEIDI test. Middle plot, eQTL results in blood for three probes, ILMN_1691693, ILMN_1797428, ILMN_1699599 **(A)** and ENSG00000160856 **(B)** probes, tagging *FCRL3/FCRH3*. Bottom plot, location of genes tagged by the probes. Highlighted in maroon indicates probes that pass SMR threshold. *FCRL3*, Fc Receptor Like 3; *FCRH3*, Fc Receptor Homolog 3; AITD, autoimmune thyroid disease; GWAS, genome-wide association studies; SMR, summary-data-based Mendelian randomization; HEIDI, heterogeneity in dependent instruments; eQTL, expression quantitative trait loci.

**Figure 4:**
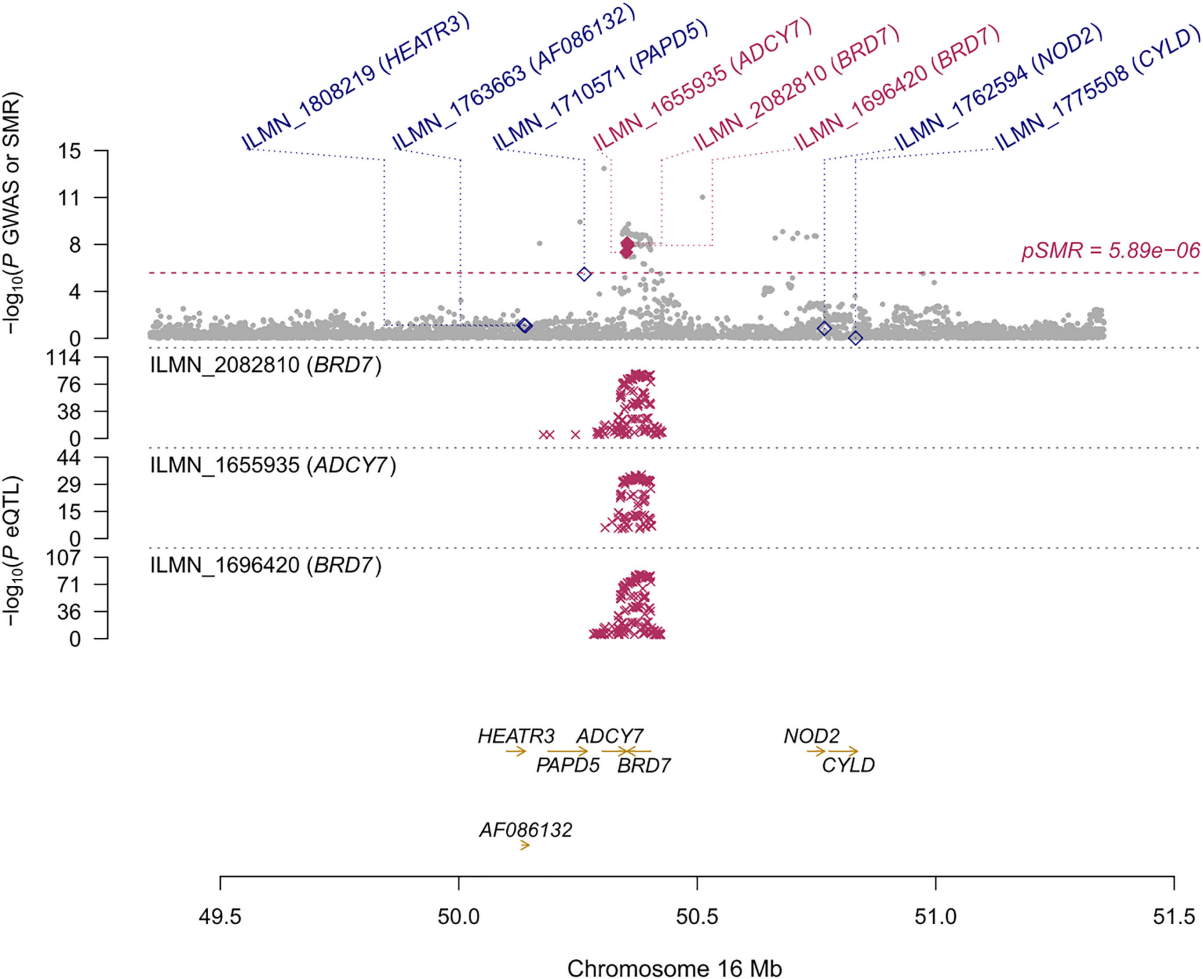
Prioritizing genes around *BRD7* in association with AITD in blood. Top plot, gray dots represent the -log 10 (*P* values) for SNPs from the GWAS of AITD, and rhombuses represent the -log 10 (*P* values) for probes from the SMR test with solid rhombuses indicating that the probes pass HEIDI test and hollow rhombuses indicating that the probes do not pass the HEIDI test. Middle plot, eQTL results in blood for two probes, ILMN_2082810, and ILMN_1696420 tagging *BRD7*. Bottom plot, location of genes tagged by the probes. Highlighted in maroon indicates probes that pass SMR threshold. *BRD7*, Bromodomain Containing 7; AITD, autoimmune thyroid disease; GWAS, genome-wide association studies; SMR, summary-data-based Mendelian randomization; HEIDI, heterogeneity in dependent instruments; eQTL, expression quantitative trait loci.

We found 71 probes that were significantly associated with AITD using CAGE, Westra, and GTEx eQTL summarized data (**Table 2**). Using AITD GWAS summarized data, we found a few probes tagging *FCRL3/FCRH3* (β [SE] = -0.055 [0.011], *P*_SMR_ = 2.05 ×10^-7^, *P*_HEIDI_ = 0.350, CAGE eQTL study; β [SE] = -0.077 [0.015], *P*_SMR_ = 2.03 ×10^-7^, *P*_HEIDI_ = 0.082, Westra eQTL study; β [SE] = -0.158 [0.032], *P*_SMR_ = 7.57 ×10^-7^, *P*_HEIDI_ = 0.089, GTEx eQTL study) (**Figures 3A, B** **)** and *BRD7* (β [SE] = 0.096 [0.017], *P*_SMR_ = 2.44 ×10^-8^, *P*_HEIDI_ = 0.078, CAGE eQTL study; β [SE] = 0.157 [0.029], *P*_SMR_ = 3.87 ×10^-8^, *P*_HEIDI_ = 0.082, Westra eQTL study; β [SE] = 0.102 [0.019], *P*_SMR_ = 3.35 ×10^-8^, *P*_HEIDI_ = 0.105, GTEx eQTL study), that showed strong pleiotropic association with AITD (**Figure 4**). Particularly, the most significant pleiotropic associations with AITD were detected on *CYP21A2* (β [SE] = -0.339 [0.040], *P*_SMR_ = 1.41 ×10^-17^, *P*_HEIDI_ = 0.079, GTEx eQTL study).

**Table 2:** Significant genes showing pleiotropic association with AITD in blood.

eQTL study	CHR	Gene	topSNP	p_GWAS	p_eQTL	Beta	SE	p_SMR	p_HEIDI	nsnp_HEIDI
CAGE	1	*RERE*	rs301802	7.40E-12	8.58E-116	0.097	0.015	5.32E-11	0.139	20
	1	*INPP5B*	rs28525112	8.20E-09	2.24E-100	-0.088	0.016	2.64E-08	0.193	20
	1	*FCRH3*	rs3761959	1.40E-07	2.16E-222	-0.055	0.011	2.05E-07	0.350	20
	1	*TNFRSF18*	rs75972122	1.00E-07	8.37E-43	-0.129	0.026	6.86E-07	0.102	20
	1	*YRDC*	rs36084352	6.20E-10	1.16E-14	0.270	0.056	1.38E-06	0.321	12
	1	*TCEB3*	rs671565	2.10E-07	4.98E-38	0.138	0.029	1.47E-06	0.228	20
	1	*UTP11L*	rs35129114	1.00E-08	9.46E-16	-0.237	0.051	3.08E-06	0.319	12
	2	*CCL20*	rs1811711	2.20E-08	1.62E-18	0.195	0.041	2.37E-06	0.154	10
	6	*CUTA*	rs4231	1.60E-14	2.96E-28	0.208	0.033	2.96E-10	0.263	20
	6	*NEU1*	rs3117573	1.40E-73	1.75E-08	0.899	0.167	7.39E-08	0.572	15
	6	*LST1*	rs116646885	1.10E-17	6.84E-10	0.387	0.077	5.57E-07	0.070	8
	6	*PHF1*	rs11759285	1.40E-12	1.76E-11	0.319	0.065	1.08E-06	0.070	5
	7	*NFE2L3*	rs916963	4.50E-08	2.56E-154	-0.065	0.012	8.47E-08	0.167	20
	7	*FOXK1*	rs7784748	5.50E-08	4.47E-32	-0.152	0.031	8.01E-07	0.487	10
	7	*FSCN1*	rs4415230	1.70E-07	1.97E-22	-0.191	0.042	4.07E-06	0.064	20
	8	*MSRA*	rs9329221	8.50E-10	4.58E-57	-0.126	0.022	1.03E-08	0.104	20
	8	*RAB2A*	rs2875974	8.80E-09	4.74E-51	-0.129	0.024	7.77E-08	0.056	20
	10	*PLEKHA1*	rs11200594	1.30E-08	1.78E-44	0.133	0.025	1.38E-07	0.128	20
	11	*CCDC88B*	rs479552	5.40E-09	1.45E-126	-0.080	0.014	1.44E-08	0.092	20
	11	*IFITM3*	rs10902121	9.10E-07	8.93E-118	0.067	0.014	1.57E-06	0.234	20
	11	*C11orf10*	rs174541	3.60E-07	1.30E-42	0.121	0.025	1.84E-06	0.970	20
	12	*SUOX*	rs7302200	8.90E-12	9.84E-25	0.208	0.037	1.32E-08	0.092	20
	16	*BRD7*	rs11644259	6.60E-09	1.59E-91	0.096	0.017	2.44E-08	0.078	20
	16	*ADCY7*	rs9934775	5.70E-09	3.15E-35	0.162	0.031	1.35E-07	0.076	14
	16	*CMTM3*	rs8051710	5.50E-07	6.09E-59	0.101	0.021	1.72E-06	0.870	20
	16	*GPR114*	rs6499882	2.20E-07	2.68E-25	0.164	0.035	3.53E-06	0.290	12
	16	*ZDHHC1*	rs45483293	4.80E-08	2.83E-18	-0.202	0.044	3.72E-06	0.270	20
	18	*CD226*	rs763361	5.10E-11	2.91E-50	0.144	0.024	1.85E-09	0.430	20
	19	*IRF3*	rs12104272	9.40E-11	7.67E-49	0.150	0.025	3.11E-09	0.156	20
	22	*TEF*	rs4822025	1.40E-09	1.02E-27	0.172	0.033	1.19E-07	0.200	20
GTEx v8	1	*INPP5B*	rs36084352	6.20E-10	1.18E-80	-0.112	0.019	4.05E-09	0.922	20
	1	*FCRL3*	rs3761959	1.40E-07	3.56E-47	-0.158	0.032	7.57E-07	0.589	20
	2	*CCL20*	rs1811711	2.20E-08	3.27E-24	0.159	0.032	9.57E-07	0.379	7
	3	*KIAA1524*	rs55943732	1.10E-07	4.75E-22	0.213	0.046	3.28E-06	0.392	12
	5	*CAMK4*	rs114378220	2.70E-11	6.82E-18	0.496	0.094	1.36E-07	0.228	5
	6	*CYP21A2*	rs3134971	4.50E-90	4.34E-21	-0.339	0.040	1.41E-17	0.079	20
	6	*HLA-W*	rs10947091	9.00E-20	1.92E-13	0.251	0.044	1.07E-08	0.121	13
	6	*HLA-G*	rs13211218	3.20E-19	1.13E-11	0.278	0.051	6.26E-08	0.227	18
	6	*CUTA*	rs78306789	6.50E-14	3.90E-10	0.988	0.206	1.55E-06	0.264	15
	7	*GIGYF1*	rs314294	9.50E-10	4.82E-17	0.305	0.062	7.68E-07	0.262	20
	8	*LRRC6*	rs1895807	4.80E-07	1.10E-114	0.072	0.015	8.86E-07	0.085	20
	8	*ALG1L13P*	rs10087493	1.40E-13	6.23E-09	0.499	0.109	4.90E-06	0.101	20
	9	*PKN3*	rs13289095	4.70E-07	1.96E-65	0.093	0.019	1.35E-06	0.320	20
	11	*AP003774.1*	rs479777	6.10E-09	1.58E-92	0.065	0.012	2.25E-08	0.654	20
	16	*BRD7*	rs11644259	6.60E-09	1.17E-72	0.102	0.019	3.35E-08	0.105	20
	16	*ATP6V0D1*	rs11860837	3.70E-09	1.78E-41	0.471	0.087	6.54E-08	0.097	20
	16	*ADCY7*	rs34222100	5.00E-09	1.38E-36	0.248	0.047	1.12E-07	0.380	12
	16	*CMTM3*	rs3743719	3.70E-07	1.58E-29	0.192	0.042	3.57E-06	0.466	20
	17	*ACAP1*	rs61759532	1.30E-15	1.72E-46	0.309	0.044	2.95E-12	0.243	20
	18	*DOK6*	rs12969657	4.10E-11	2.22E-14	-0.234	0.047	5.92E-07	0.615	14
westra	1	*RERE*	rs301806	1.90E-11	5.92E-165	0.122	0.019	7.02E-11	0.055	20
	1	*FCRL3*	rs945635	1.50E-07	1.57E-277	-0.077	0.015	2.03E-07	0.082	20
	1	*TCEB3*	rs519202	2.80E-07	2.09E-52	0.159	0.033	1.13E-06	0.553	20
	1	*SF3A3*	rs4072980	1.30E-06	1.10E-190	-0.082	0.017	1.79E-06	0.065	20
	2	*GLS*	rs13414554	2.00E-08	3.59E-18	-0.294	0.062	2.42E-06	0.109	20
	6	*BACH2*	rs10944479	5.20E-34	5.52E-13	0.790	0.127	5.55E-10	0.998	6
	6	*SESN1*	rs12197912	4.50E-07	1.29E-84	-0.124	0.025	1.03E-06	0.661	10
	7	*FOXK1*	rs7784748	5.50E-08	5.63E-44	-0.186	0.037	4.16E-07	0.821	6
	7	*FSCN1*	rs4415230	1.70E-07	3.53E-20	-0.280	0.062	5.45E-06	0.086	20
	7	*ZNF282*	rs12530946	2.60E-06	1.59E-47	0.152	0.034	7.80E-06	0.450	20
	8	*MSRA*	rs9329221	8.50E-10	6.18E-94	-0.144	0.025	4.12E-09	0.224	20
	8	*DKFZP761P0423*	rs13274039	5.20E-11	1.09E-11	0.459	0.097	2.35E-06	0.156	20
	8	*RAB2A*	rs671275	1.10E-06	2.82E-48	-0.155	0.034	3.80E-06	0.162	20
	11	*C11orf10*	rs174574	2.40E-07	4.54E-56	0.156	0.032	9.16E-07	0.685	20
	11	*CD44*	rs8193	6.00E-12	1.39E-09	0.535	0.118	5.47E-06	0.174	20
	13	*GPR18*	rs1923894	2.20E-07	1.11E-21	0.259	0.057	5.21E-06	0.056	20
	16	*BRD7*	rs8059133	7.30E-09	7.84E-70	0.157	0.029	3.87E-08	0.082	12
	16	*PAPD5*	rs4785412	5.50E-09	2.17E-21	0.291	0.059	6.72E-07	0.188	9
	16	*CCDC102A*	rs2107238	2.00E-07	6.46E-27	0.228	0.049	2.87E-06	0.084	16
	18	*CD226*	rs763361	5.10E-11	2.43E-50	0.209	0.035	1.84E-09	0.300	20
	19	*IRF3*	rs7251	3.70E-11	1.87E-109	0.144	0.023	2.29E-10	0.074	17

p_GWAS is the P value for the top associated cis-eQTL in the GWAS analysis and p_eQTL is the P value of the top associated cis-eQTL in the eQTL analysis, and Beta is the estimated effect size of SMR, SE is the corresponding standard error, p_SMR is the P value for SMR analysis, p_HEIDI is the P value for the HEIDI test and nsnp is the number of SNPs involved in the HEIDI test.

#### 3.6.3 SMR analysis in thyroid

**Figure 5:**
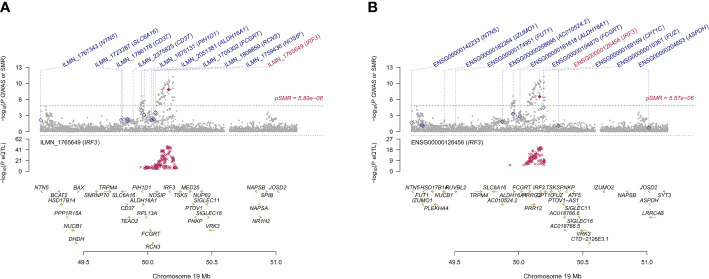
Prioritizing genes *IRF3* in association with AITD in blood **(A)** and thyroid **(B)**. Top plot, gray dots represent the -log 10 (*P* values) for SNPs from the GWAS of AITD, and rhombuses represent the -log 10 (*P* values) for probes from the SMR test with solid rhombuses indicating that the probes pass HEIDI test and hollow rhombuses indicating that the probes do not pass the HEIDI test. Middle plot, eQTL results in blood. Bottom plot, location of genes tagged by the probes. Highlighted in maroon indicates probes that pass SMR threshold. *IRF3*, Interferon Regulatory Factor 3; AITD, autoimmune thyroid disease; GWAS, genome-wide association studies; SMR, summary-data-based Mendelian randomization; HEIDI, heterogeneity in dependent instruments; eQTL, expression quantitative trait loci.

The GTEx eQTL summary data revealed twenty-four genes that were pleiotropically correlated with AITD in thyroid tissue (**Table 3**). *NR3C2* displayed the most significant pleiotropic associations with AITD (β [SE] = 0.200 [0.021], *P*_SMR_ = 6.88 ×10^-21^, *P*_HEIDI_ = 0.367), then *TPO* (β [SE] = -0.208 [0.025], *P*_SMR_ = 8.91 ×10^-17^, *P*_HEIDI_ = 0.886) and *RNASET2* (β [SE] = 0.465 [0.061], *P*_SMR_ = 3.73 ×10^-14^, *P*_HEIDI_ = 0.107). Of note, *IRF3* showed significant pleiotropic association not only in the thyroid but also in the blood (β [SE] = 0.307 [0.057], *P*_SMR_ = 8.41 ×10^-8^, *P*_HEIDI_ = 0.613; and β [SE] = 0.150 [0.025], *P*_SMR_ = 3.11 ×10^-9^, *P*_HEIDI_ = 0.156; β [SE] = 0.144 [0.023], *P*_SMR_ = 2.29 ×10^-10^, *P*_HEIDI_ = 0.074) (**Figures 5A, B**).

**Table 3:** Significant genes showing pleiotropic association with AITD in thyroid tissue.

eQTL study	CHR	Gene	topSNP	p_GWAS	p_eQTL	Beta	SE	p_SMR	p_HEIDI	nsnp_HEIDI
GTEx	1	*RP5-1115A15.1*	rs3795310	3.30E-09	9.20E-14	0.164	0.035	3.60E-06	0.698	16
	1	*MMEL1*	rs2100574	4.80E-07	8.42E-35	-0.104	0.022	3.17E-06	0.179	20
	1	*VAV3-AS1*	rs77475507	1.70E-51	2.38E-13	-0.327	0.050	4.38E-11	0.101	8
	2	*RP11-554J4.1*	rs1045920	4.00E-08	3.66E-54	-0.069	0.013	2.27E-07	0.200	20
	2	*TPO*	rs11675342	1.40E-21	1.17E-64	-0.209	0.025	8.91E-17	0.886	15
	3	*LRRFIP2*	rs7616105	1.30E-06	1.66E-137	0.049	0.010	2.02E-06	0.318	20
	3	*SYN2*	rs77116964	9.10E-09	4.58E-27	-0.115	0.023	3.97E-07	0.053	18
	4	*NR3C2*	rs7675216	1.40E-29	1.92E-63	0.200	0.021	6.88E-21	0.367	20
	5	*ZBED3*	rs9687206	3.20E-09	1.79E-18	-0.245	0.050	9.24E-07	0.143	20
	6	*WDR46*	rs465151	3.70E-17	7.19E-09	-0.659	0.138	1.85E-06	0.307	11
	6	*LINC00271*	rs12181879	2.00E-08	1.08E-20	0.245	0.051	1.52E-06	0.060	20
	6	*UHRF1BP1*	rs9296128	3.90E-08	7.91E-28	-0.148	0.030	9.10E-07	0.061	20
	6	*PDE10A*	rs2983511	6.00E-09	2.21E-21	-0.155	0.031	7.05E-07	0.504	12
	6	*MSH5*	rs532086	1.70E-55	1.92E-08	1.085	0.205	1.22E-07	0.636	20
	6	*PPP1R18*	rs3131041	2.30E-40	7.51E-09	0.774	0.146	1.16E-07	0.374	20
	6	*FGFR1OP*	rs6456143	5.10E-26	1.44E-20	-0.402	0.058	3.06E-12	0.061	20
	6	*RNASET2*	rs2769352	3.20E-30	5.09E-24	0.465	0.061	3.73E-14	0.107	20
	7	*RP4-800G7.2*	rs7790005	1.80E-07	6.32E-23	-0.179	0.039	3.98E-06	0.599	16
	8	*RP11-419I17.1*	rs11998678	8.00E-10	9.51E-13	0.178	0.038	3.21E-06	0.068	20
	8	*ALG1L13P*	rs2980439	8.40E-15	1.18E-09	0.246	0.051	1.68E-06	0.052	20
	8	*RAB2A*	rs2875967	2.30E-09	1.48E-19	-0.391	0.078	6.17E-07	0.218	20
	13	*RAB20*	rs2479434	1.30E-06	6.28E-67	0.080	0.017	3.15E-06	0.871	20
	16	*HSD11B2*	rs9936320	6.10E-09	5.81E-23	-0.205	0.041	5.47E-07	0.070	20
	19	*IRF3*	rs2304205	6.60E-11	6.90E-21	0.307	0.057	8.41E-08	0.613	20

p_GWAS is the P value for the top associated cis-eQTL in the GWAS analysis and p_eQTL is the P value of the top associated cis-eQTL in the eQTL analysis, and Beta is the estimated effect size of SMR, SE is the corresponding standard error, p_SMR is the P value for SMR analysis, p_HEIDI is the P value for the HEIDI test and nsnp is the number of SNPs involved in the HEIDI test.

### 3.7 The result of PheWAS

**Figure 6:**
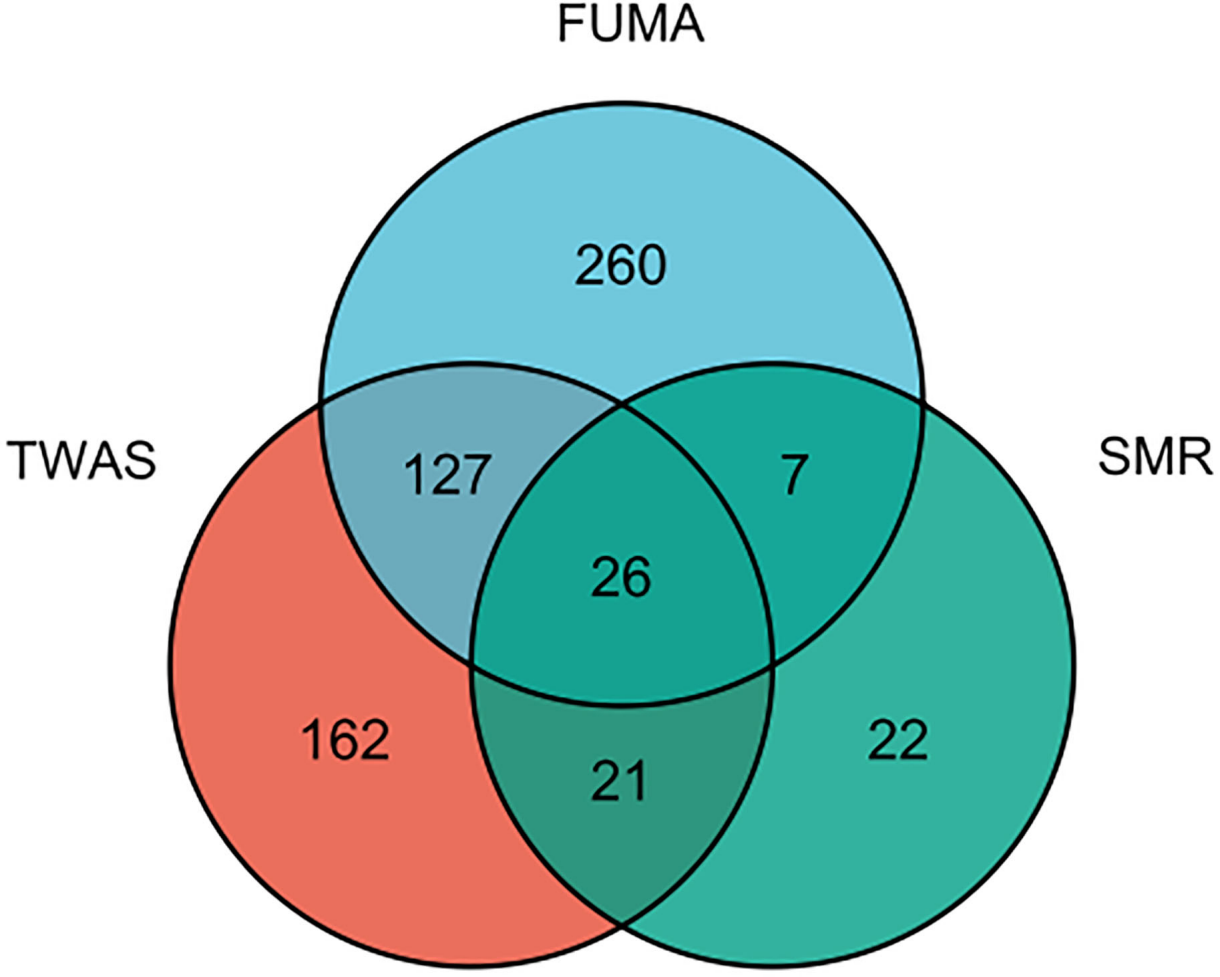
The result of an integration TWAS, FUMA and SMR analysis.

Since plenty of genes were associated with AITD, we selected 26 genes that were simultaneously present in the result of TWAS, FUMA and SMR analysis (**Figure 6** **;** **Supplementary Table S10**). We performed a PheWAS and identified the risk of other associated or co-morbid phenotypes for AITD-related genes, which included immunological traits (e.g., granulocyte percentage of myeloid white cells); connective tissue traits (e.g., rheumatoid arthritis); cardiovascular traits (e.g., resting heart rate). The top ten phenotypes of 26 genes are presented in **Supplementary Table S11**.

## 4 Discussion

AITD is a prevalent autoimmune condition that affects millions of people all over the world (2). Although recent GWAS have been effectively identifying a few risk loci for AITD, the functional significance of these correlations has remained unknown due to the difficulties in interpreting LD and inferring causality (24). TWAS takes full advantage of eQTL expression reference panels that comprise expression and genotype data to seek gene-trait correlations from GWAS datasets (10, 12, 16). The current study was the first to attempt an AITD TWAS by applying the summary statistics of 755,406 AITD individuals (30,234 cases, and 725,172 controls) from the most recent ATID GWAS to reveal the genetic components of AITD gene expression through an extensive exploration of different tissue types, and intensive characterization of identified associations representing pivotal transcriptome changes.

Here, several key findings were emphasized. First, we identified 330 genes significantly associated with AITD, the majority of which were novel (296/330). 118 of the 330 significant features were independently associated with AITD, implying that the majority of the detected features are dependent on LD and correlated predicted expression of neighboring genes. Following that, based on a set of subsequent analyses, we discovered 9 correlations with AITD due to their significant TWAS signal, colocalization (PP4), and PIP. Furthermore, using the FUMA approach, prioritized 583 unique genes from 128 loci through the MAGMA tool. Moreover, utilizing SMR analysis, we totally identified 95 genes that showed pleiotropically associated with AITD in blood and thyroid tissues. Noteworthily, we finally obtained 26 key susceptibility genes by integrating the results of TWAS, FUMA, and SMR methods. When compared to the aforementioned meta-analysis, 12 of these 26 key genes had previously been reported, while 14 were novel (14).

We confirmed previously reported genes such as *BACH2*, *TPO*, *RNASET2*, *IRF*, etc. *BACH2* is a transcriptional repressor in the *BACH* family of basic leucine zipper transcription factors. The *BACH2* protein is generated throughout B-cell maturation and is important in the development and function of both the innate and adaptive immune systems (25, 26). Thyroid peroxidase (*TPO*) encoded protein functions as an enzyme and is essential for thyroid gland function. AITD-associated loci, including *TPO*-rs11675434, and *BACH2*-rs10944479, were discovered in a GWAS meta-analysis including 18,297 individuals, with 1,769 *TPO*Ab-positives and 16,528 *TPO*Ab-negatives for *TPO*Ab-positivity and in 12,353 individuals for *TPO*Ab serum levels, with replication in 8,990 individuals (27). *RNASET2* is a ribonuclease that is important in the innate immune response by identifying and degrading RNAs from microbial pathogens that are then detected by *TLR8* (28). The gene *RNASET2* has been suggested as a putative risk hotspot for GD in GWASs (8). An emerging longitudinal, case-control study suggested that *RNASET2* was considerably overexpressed in GD patients in comparison to healthy controls (29). However, since the evidence is questionable due to the limited sample size, replication is necessary. The gene *IRF3* showed an observably pleiotropic relationship with AITD both in blood and the thyroid. *IRF3*, encoding a member of the interferon regulatory transcription factor (*IRF*) family, functions as a key transcriptional regulator of type I interferon-dependent immune responses, which are crucial in the innate immune response (30). Previous GWASs have also identified an association between *IRF3* and various autoimmune diseases such as RA, T1DM, and SLE (31–33). Additionally, coinciding with an original meta-analysis of GWAS conducted by Saevarsdottir et al. (14), we found *FCRL3* was significantly associated with AITD. According to the predicted molecular structure, *FCRL3* may function by encoding a membrane protein that conveys signals into cells (34). Moreover, previous studies suggested that *FCRL3* might be a risk gene for RA (35, 36). The findings above, together with ours, indicated that *FCRL3* plays a significant role in the immune system, emphasizing its enormous potential as a prospective target for AITD treatment and prevention.

Among these 14 novel genes identified, *CYP21A2*, *MSH5*, *PPP1R18*, and *MMEL1* are found in or near the human major histocompatibility complex (MHC) region on chromosome 6, where these genes play a crucial function in immune response and immunological regulation and are implicated in several inflammatory and autoimmune diseases (37–42). Previous GWASs identified plenty of genetic variants in the MHC region correlated with AITD (8, 9). In blood, we found that *CYP21A2* had the most significant pleiotropic associations with AITD. *CYP21A2*, as well as *MSH5*, *PPP1R18*, and *NEU1* have been demonstrated by previous GWASs associated with other autoimmune disorders such as RA, ankylosing spondylitis (AS), T1DM, and SLE (31–33, 43, 44). In addition, the three genes *MMEL1*, *CUTA*, and *CCDC88B* were found that have significant relation with RA (32), the gene *SUOX* with RA as well as T1DM (31, 32), and *UHRF1BP1* with SLE (33). Thyroid disorders have frequently occurred in patients with systemic autoimmune conditions including RA and SLE (45, 46). For example, genetic variations were presented in a research of 35 families with numerous cases of SLE accompanied by AITD, in which a gene susceptibility locus was detected in 5q14.3-q15, the major susceptibility locus for SLE, also observed in AITD (47). To some extent, such a result suggested a potential genetic link between SLE and AITD despite the small samples. The gene *BRD7*, in particular, was found to be significantly associated with AITD. *BRD7* is a member protein of the bromodomain-containing protein family and plays a crucial role in the pathogenesis of cancers and the regulation of inflammation, metabolism, and obesity (48–50). In addition, a cross-sectional study revealed that obesity raises the risk of AITD by affecting a loop involving leptin (51). This is the first study to identify a connection between the gene *BRD7* and AITD; moreover, published studies on the correlation between *BRD7* and other autoimmune disorders are scarce, and more study is urgently needed.

Additionally, gene-set analysis indicated that the top five predominant pathways correlated with AITD were positive regulation of immune system process, regulation of immune system process, lymphocyte activation, T cell activation, and immune system development. There are some commonalities between AITD and other autoimmune diseases. Huber, A et al. (52) believed that all currently identified genes associated with both AITD and T1DM are involved in immune regulation, specifically in presenting antigenic peptides to T cells. Further, activated lymphocytes are often involved in the progression of autoimmune diseases such as SLE and RA (53), which has also been proven by Burbano, C et al. (54).

These findings above underline the transcriptomic alterations in AITD that probably play essential roles in disease etiology and pathogenesis, attributed to the same genetic variations as the disease and determinants, resulting in transcriptomic alterations in gene expression and traits. Such outcomes on key genes and gene-sets for AITD can guide future drug target research as well as candidate gene exploration in animal or cell research, where the consequences of molecular and its pathway changes can be easier to be explored by inducing gene knock-down or up-regulation.

Although our findings were promising, several major limitations need to be addressed. First, since only European individuals were involved in this investigation, the findings may not apply to other ethnicities. More research is required to verify the current results in specific ethnic groups, such as Asians. In addition, the validity of our findings was constrained owing to the relatively limited sample sizes of available gene expression references, suggesting that larger samples are urgently required. Furthermore, because the GWAS samples had a wide range of AITD definitions (GD or HT), future study is required to examine how restricted phenotyping influences findings at the transcriptomic level based on more accurate definitions.

In summary, we present evidence for extensive alterations in AITD at a transcriptomic level. Our study can identify new associations and characterize the transcriptomic alterations that currently recognized risk factors undergo. We also identified several genes that are likely to be essential in AITD. These results indicate that the genetic component of gene expression is crucial in AITD.

## Data availability statement

The original contributions presented in the study are included in the article/**Supplementary Material**. Further inquiries can be directed to the corresponding authors.

## Author contributions

Conceptualization: XL. Methodology: XL. Formal analysis and investigation: XL and YM. Writing - original draft preparation: XL. Writing - review and editing: XL, CL, WL, QF. Funding acquisition: QZ. Resources: XL and YM. Supervision: QZ and all authors commented on previous versions of the manuscript. All authors read and approved the final manuscript.

## Supplementary material

Supplementary Figure 1 Summary of genomic risk loci based on AITD GWAS. Y axis, Genomic risk loci are displayed by the ‘chromosome: start position-end position’. X axis, Histograms from left to right depict the size of the genomic loci, the number of candidate SNPs, mapped genes by positional mapping and eQTL mapping in the genomic loci, and the number of identified genes located within the genomic loci, respectively.
