# Supplementary figures and images for: Identification of multiple novel susceptibility genes associated with autoimmune thyroid disease
Source: Front Immunol. 2023 May 1;14:1161311. doi: 10.3389/fimmu.2023.1161311 (PMC10183592; doi:10.3389/fimmu.2023.1161311)

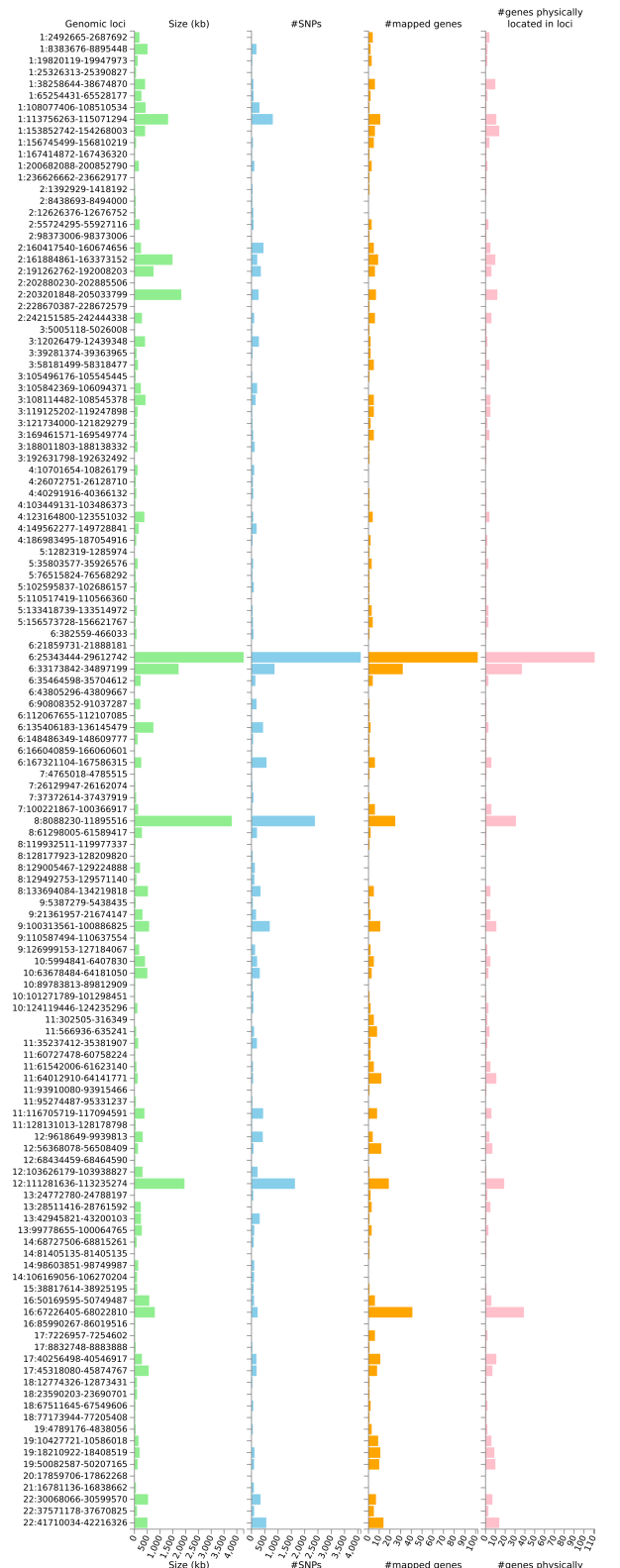

Supplement: Supplementary Figure 1 — Summary of genomic risk loci based on AITD GWAS. Y axis, Genomic risk loci are displayed by the ‘chromosome: start position-end position’. X axis, Histograms from left to right depict the size of the genomic loci, the number of candidate SNPs, mapped genes by positional mapping and eQTL mapping in the genomic loci, and the number of identified genes located within the genomic loci, respectively. [file Image_1.pdf]
